# Ionic Liquids and Calcium Oxide Grafted with Allylmalonic Acid Applied to Support the Peroxide Crosslinking of an Ethylene–Propylene Copolymer

**DOI:** 10.3390/ma13153260

**Published:** 2020-07-22

**Authors:** Magdalena Maciejewska

**Affiliations:** Institute of Polymer and Dye Technology, Lodz University of Technology, Stefanowskiego Street 12/16, 90-924 Lodz, Poland; magdalena.maciejewska@p.lodz.pl

**Keywords:** coagent, allylmalonic acid, ionic liquid, calcium oxide, elastomer, peroxide crosslinking, ethylene–propylene copolymer

## Abstract

Nanosized calcium oxide (CaO) featuring a surface grafted with allylmalonic acid (ALA) was used to increase the efficiency of the peroxide crosslinking of an ethylene–propylene copolymer (EPM) filled with silica nanoparticles. In this study, 1-butyl-3-methylimidazolium ionic liquids (ILs) with different anions were applied to improve the dispersion of CaO/ALA and silica nanoparticles in the EPM copolymer, as well as to catalyze the interfacial crosslinking reactions. In this article, we discuss the effects of CaO/ALA and ILs on the curing characteristics, vulcanization temperature, crosslink density, mechanical properties, and thermal stability of EPM, as well as the resistance of EPM to weather aging. The CaO/ALA with ILs reduced the vulcanization time of the rubber compounds without a significant effect on the vulcanization temperature. Their application resulted in an increased vulcanizate crosslink density, as well as improved tensile strength compared to the pure peroxide system. The influence of 1-butyl-3-methylimidazolium ILs on EPM vulcanization and performance depends on the anion present in the molecules of the ionic liquid. The most active IL seems to be that with the tetrafluoroborate anion.

## 1. Introduction

The vulcanization of rubber is one of the most important processes in elastomer technology. During this process, crosslinking reactions occur, resulting in useful materials that possess the required physical properties, such as high tensile or tear strengths, a low compression set, recoverable elongation, and improved dynamic performance. The final properties of the crosslinked material depend primarily on the quantity and quality of the crosslinks formed in the resulting elastomer network [1]. Many types of vulcanization systems have been used industrially for several years. The choice of a specific vulcanization system or crosslinker depends primarily on the type of rubber (saturated, unsaturated, or containing special functional groups), the desired vulcanization conditions (temperature and optimal vulcanization time), and the required physical properties of the final vulcanizates. The most popular crosslinking agents are sulfur in the presence of activators and vulcanization accelerators for unsaturated rubbers [2,3], organic peroxides for saturated polymers [1,4], and metal oxides for rubbers containing functional groups, such as carboxyl or chlorosulfone [5,6,7]. However, peroxides are capable of vulcanizing both unsaturated and saturated elastomers, as well as fluoroelastomers and silicones [8].

Peroxide vulcanizates are resistant to thermal aging and ozone. On the other hand, their poor mechanical and dynamic properties compared to sulfur-crosslinked elastomers limit their industrial applications. In order to enhance the mechanical performance, crosslink density, and vulcanization efficiency of peroxide-crosslinked rubber compounds, special auxiliaries called coagents are used [9]. This role can be played by multifunctional vinyl compounds, which are able to easily and effectively react with free radicals formed in the initial stage of peroxide curing. The presence of double bonds makes the coagents molecules capable of undergoing addition or polymerization reactions during polymer curing. Owing to these reactions, coagents molecules are incorporated into the crosslinked elastomer network. On the other hand, functional groups of coagents can form additional non-covalent linkages, which are ionic or complex crosslinks [9]. Regarding their influence on the cure rate, coagents are grouped into two categories. The first type includes acrylates, methacrylates, and maleimides. This group of coagents increases the cure rate and may lead to scorching, which is a serious disadvantage because it reduces the safety of the processing of rubber compounds and hinders the formation of the final rubber products. Coagents of the second type improve crosslinking efficiency without affecting the cure rate or adding scorch. This group of coagents includes polybutadienes and allylated compounds like triallyl cyanurate, triallyl isocyanurate, and diallyl phthalate [10]. Some coagents, such as zinc acrylate or methacrylate, are used to create ionic crosslinks in the crosslinked elastomer network. These are labile ionic links that allow for considerable chain slippage and reformation of bonds, resulting in the increased ability of the material to relax stress. Consequently, its mechanical properties, such as tensile strength and tear resistance, are greatly improved [11,12].

Another approach to improve the efficiency of rubber peroxide crosslinking and the mechanical properties of the vulcanizates are hybrid coagents consisting of an inorganic core and an organic shell, which were reported in our previous works [13,14,15]. These coagents are based on nanosized metal oxides (ZnO, CaO, MgO) and layered minerals (hydrotalcite, boehmite) modified with unsaturated carboxylic acids, which possess easily cleavable protons and double bonds, that are readily accessible for reactions with free radicals or polymer chains. Unsaturated acids are grafted onto the inorganic core during the chemical modification process in acetone. Depending on their structure, these hybrid coagents can form ionic crosslinks of a labile nature, which are able to slip on the surfaces of inorganic core particles (metal oxide or layered minerals) under the influence of an external force. As a result, it is possible to increase the material’s susceptibility to stress relaxation. The problem of using coagents based on nanosized metal oxides is the agglomeration of nanoparticles in the elastomer matrix. Agglomerates reduce the surface of contact between the functional groups on the coagent particles and elastomer chains, which is crucial in crosslinking reactions. Therefore, it is necessary to develop substances that improve the dispersion degree of coagent nanoparticles in the elastomer matrix. These potential dispersing agents can be ionic liquids (ILs) that, owing to their catalytic activity in interfacial reactions (including crosslinking reactions), can also increase the efficiency of crosslinking [16].

Recently, ILs have been widely applied in polymers technology [17,18]. The most important advantages of ILs, that determine their use as additives to elastomer composites are high temperature of thermal degradation, non-flammability, and non-volatility [17]. No less important for some applications is high ionic conductivity resulting from the ionic structure o ILs [19,20].

Considering applications in polymer technology, ILs are commonly used as “green” solvents for various types of polymerizations, including free radical polymerization and living cationic polymerization [21,22,23]. They have also been used as very effective solvents of polymers, such as cellulose [24] or starch [25]. Due to the high ionic conductivity and good electrochemical stability, ILs have been successfully applied as an alternative to commonly used lithium salts in solid polymer electrolytes (SPEs) based on elastomers. Alkylimidazolium salts of tetrafluoroborate or bis(trifluoromethylsulfonyl)imide were used as ion sources for SPEs based on acrylonitrile–butadiene elastomer (NBR) to produce flexible and mechanically stable SPEs [26,27]. Alkylimidazolium and pyrrolidinium ILs were applied by Likozar [28] to produce SPEs based on hydrogenated acrylonitrile–butadiene elastomer (HNBR) filled with hydroxy-functionalized multi-walled carbon nanotubes. SPEs were prepared by melt compounding of the nanotubes in the elastomer, curing of the rubber compounds and immersion of the elastomer composites in the ionic liquid.

Recently, ILs have been applied to improve the distribution of particles in rubber composites [29,30], especially for silica, clays, and carbon fillers, especially carbon nanotubes [30,31,32]. Filler modification with ILs was reported to strengthen the interactions between a filler and an elastomer matrix and consequently promote the uniform distribution of filler particles in the elastomer. As a result, the mechanical performance of various elastomer composites was effectively improved [30,33,34]. ILs have also been used to increase the dispersion degree of vulcanization activator such as nanosized zinc oxide in styrene–butadiene (SBR) rubber. This resulted in increasing the rate of vulcanization, lowering the temperature of vulcanization and increasing the concentration of crosslinks in the crosslinked elastomer network [35]. 

Catalytic activity in interfacial reactions and dispersing action of ILs have been used successfully in the crosslinking of elastomers. For example, a novel ionic liquid-crosslinked flexible polyurethane elastomer was fabricated using tris(2-hydroxyethyl)methylammonium methylsulfate as crosslinker [36]. Polyurethane elastomers crosslinked with ionic liquid demonstrated significantly higher tensile strengths and elongation at break compared with conventional thermoplastic polyurethane elastomers, as well as improved high-temperature oil resistance. The accelerating action of alkylimidazolium ILs with various anions on the crosslinking process was reported for carboxylated acrylonitrile–butadiene elastomer (XNBR) filled with hydrotalcite [37] and NBR filled with silica [38]. The accelerating effect of ILs resulted in the reduction in the scorch time and the curing time of rubber compounds. Additionally, vulcanizates with ILs exhibited higher crosslink density compared to those without ionic liquid. 

In this work, nanosized CaO with its surface grafted with an unsaturated acid, such as allylmalonic acid (ALA), was applied as a coagent for the peroxide crosslinking of an ethylene–propylene copolymer (EPM). A hybrid coagent consisting of an inorganic core and an organic shell was achieved in this way. A thermal analysis and mechanical methods were employed to determine the effects of CaO/ALA and ILs on the crosslinking characteristics and performance of EPM composites. ILs, such as 1-butyl-3-methylimidazolium bromide, chloride, tetrafluoroborate, and hexafluorophosphate, were applied to improve the dispersion of CaO/ALA and nanosized silica particles in the elastomer matrix. Additionally, ILs were reported to catalyze interfacial reactions [35,39,40]. Thus, they can be assumed to play the same role in peroxide crosslinking. 

ILs with 1-butyl-3-methylimidazolium (Bmim) cation were chosen due to their positive influence on the vulcanization, crosslink density and performance of other elastomers, such as acrylonitrile–butadiene elastomer (NBR) and hydrogenated acrylonitrile–butadiene elastomer (HNBR), as was confirmed by our previous studies [39,41]. Furthermore, ILs with Bmim cation and different anions were reported to promote the dispersion of various fillers in the elastomer composites [34,42,43]. On the other hand, ILs with Bmim cation were observed to be more easily introduced into the rubber during the preparation of rubber compounds compared to ILs with shorter alkyl chains, which is important for technological reasons.

## 2. Materials and Methods

### 2.1. Materials

An EPM copolymer (Dutral CO 034) containing 28 wt % of propylene was obtained from Versalis (San Donato Milanese, Italy). Its Mooney viscosity was ML1+4 (125 °C):44. It was cured with dicumyl peroxide (DCP). Nanosized calcium oxide (CaO) with an average particle size <160 nm grafted with allylmalonic acid (ALA) was applied as a crosslinking coagent, except for the benchmark rubber compound in which triallyl cyanurate (TAC) was used as a commercial coagent. All these reagents were provided by Sigma-Aldrich, Darmstadt, Germany and used without purification. The structure and molar mass of the ALA are shown in Table 1. The ILs presented in Table 2 were manufactured by Ionic Liquids Technologies GmbH, Heilbronn, Germany. Silica Aerosil 380 with a specific surface area of 380 m^2^/g (Evonic Industries, Essen, Germany) was used as a filler.

### 2.2. Preparation of the CaO/ALA Coagent

ALA was grafted onto the nanosized CaO surface in the process of chemical modification using acetone as a solvent. CaO nanopowder was mixed with the solution of ALA in acetone for 30 min during ultrasonic treatment (Bandelin Sonorex Digitec DT 255, Berlin, Germany) with a frequency of 35 kHz. Then, the mixture was left for 24 hours. Acetone was evaporated the next day using a vacuum evaporator (BUCHI Labortechnik AG, Flawil, Switzerland) at 50 °C. The contents of the flask were mixed for 10 min before the evaporation process. The CaO/ALA obtained as a beige powder was dried in a vacuum drier (Memmert, Schwabach, Germany) at 60 °C for 96 hours. The quantity of ALA used for grafting was 8 g/100 g of CaO.

### 2.3. Characterization of CaO/ALA Coagent

Raw and ALA-grafted CaO was characterized using thermogravimetry (TG) with a TGA/DSC1 (Mettler Toledo, Greifensee, Switzerland) analyzer. Analyses were conducted in an inert gas atmosphere (argon, 50 mL/min) by heating the samples from 25 to 600 °C, with a heating rate of 10 °C/min. The mass losses obtained for raw and ALA-grafted CaO, were applied to calculate the content of ALA on the CaO surface, and consequently the efficiency of CaO modification. Analysis of evolved gas was performed using Setsys TG-DTA 16/18 analyzer (SETARAM Instrumentation, Caluire-et-Cuire, France) coupled to a Balzers (Pfeiffer, Aßlar, Germany) mass spectrometer.

Fourier transform infrared (FTIR) spectroscopy was used to identify impurities present in raw CaO. Measurements were carried out using a FTIR Nicolet 6700 (ThermoFisher Scientific, Waltham, MA, USA) spectrophotometer. FTIR spectrum was obtained in the range of wavenumber from 4000 to 400 cm^-1^ during 128 scans. Attenuated Total Reflectance (ATR) technique equipped with a single reflection diamond ATR crystal on ZnSe plate was used for all measurements.

### 2.4. Preparation and Characterization of EPM Compounds

EPM compounds with their compositions presented in Table 3 were prepared using a laboratory two-roll mill. After 48 hours of storage, the rheometric measurements were performed at 160 °C using an oscillating disc rheometer WG-02 (ZACH Metalchem, Gliwice, Poland). The optimal vulcanization time (t_90%_) and scorch time (t_20%_) were determined according to the standard PN-ISO 3417:1994.

The temperature and enthalpy of the EPM compound’s vulcanization were examined with a DSC1 differential scanning calorimeter (Mettler Toledo, Greifensee, Switzerland). Prior to the measurements, the samples were cooled to −60 °C using liquid nitrogen as the cooling agent. Next, the frozen samples were heated to 250 °C (heating rate of 10 °C /min) in an inert atmosphere. The mass of the test samples was approximately 10 mg.

The crosslink density (*ν_T_*) of the EPM vulcanizates was determined based on the results of equilibrium swelling in toluene. The Flory–Rehner equation [44] and the Huggins parameter of elastomer–toluen interactions given by Equation (1) [15] were used to calculate the crosslink density, where *V_r_* is the volume fraction of the elastomer in the swollen gel.

*χ* = 0.425 + 0.340 *V_r_* (1)

Furthermore, to investigate the content of ionic crosslinks (Δ*ν*) in the crosslinked elastomer network, the samples were inserted in glass weighing bottles with toluene and placed in a desiccator with saturated ammonia vapor (25% aqueous solution). Equation (2) was applied to calculate the content of ionic crosslinks, where *ν_A_* is the crosslink density determined for the vulcanizates treated with saturated ammonia vapor:

(2) $$\Delta \nu =\frac{\nu_{T}-\nu_{A}}{\nu_{T}}•100\%$$

The tensile properties of the vulcanizates were determined for dumbbell-shaped samples according to ISO-37. A Zwick Roell 1435 (Zwick Roell, Ulm, Germany) universal machine was employed to perform tensile measurements.

A TGA/DSC1 (Mettler Toledo, Greifensee, Switzerland) analyzer was employed to study the thermal decomposition of the vulcanizates. Small pieces of the vulcanizates with a mass of approximately 10 mg were placed in open ceramic crucibles (polycrystalline alumina) and were heated from 25 to 700 °C in an inert atmosphere (argon, 50 mL/min) at a heating rate of 10 °C/min. 

The weather aging of the vulcanizates was conducted for 100 hours using a CI 4000 “Xenon Arc Weather-Ometer” (Atlas, Mount Prospect, IL, USA) aging machine. During the aging process, day–night segments were repeated with the following conditions: day (duration 102 min, irradiation of 60 W/m^2^, 367 kJ, black panel temperature 80 °C, panel chamber temperature 38 °C, humidity 50%, spray); night (duration 18 min, temperature of black panel 80 °C, panel chamber temperature 38 °C, irradiation 60 W/m^2^, 64 kJ, humidity 5%, no spray). The resistance of the EPM vulcanizates to weather aging was determined following the procedure described in [27].

The dispersion of the CaO/ALA and silica particles in the EPM matrix was examined using a LEO 1530 SEM (Zeiss, Oberkochen, Germany) scanning electron microscope. Prior to the measurements, the vulcanizates were immersed in liquid nitrogen for 5 min and broken down. The surfaces of their fractures were coated with carbon and were then examined.

## 3. Results and Discussion

### 3.1. Efficiency of CaO Modification with ALA

**Figure 1:**
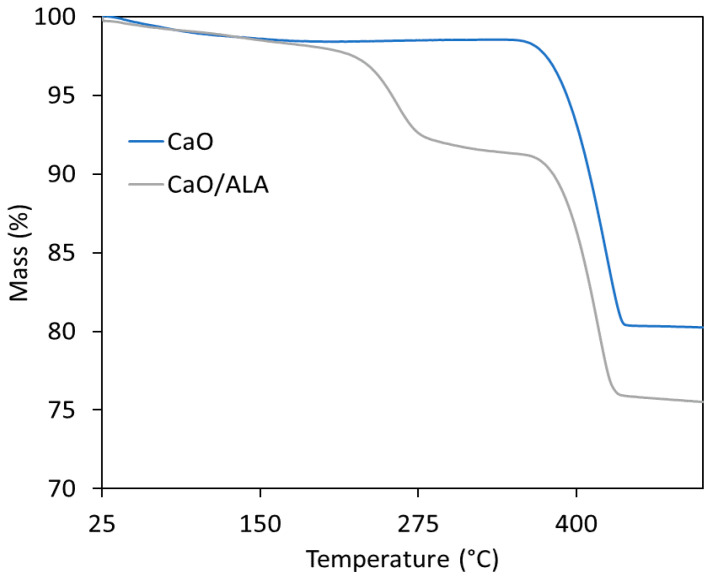
Thermogravimetric (TG) curves for pure and ALA-modified CaO.

**Figure 2:**
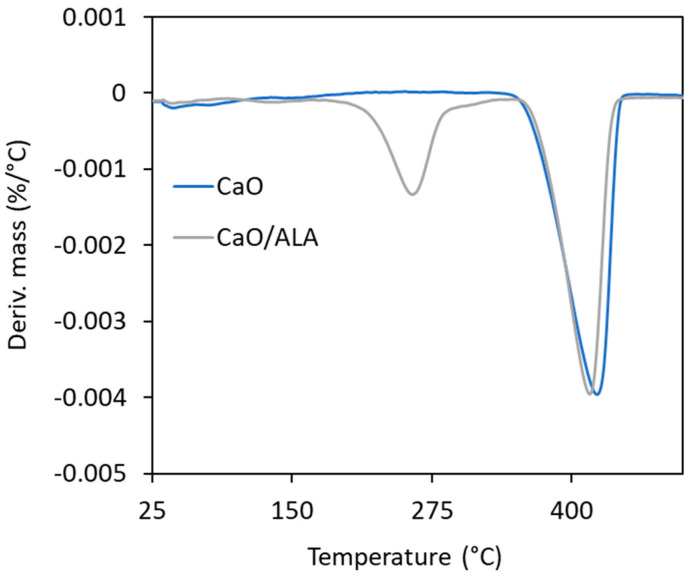
Differential thermogravimetric (DTG) curves for pure and ALA-modified CaO.

To estimate the effectiveness of CaO modification with ALA, a TG analysis of the coagent was performed (Figure 1 and Figure 2). The mass losses for pure and ALA-modified CaO were determined. Based on these results, the amount of ALA in the coagent was evaluated. The results are given in Table 4 and Table 5, whereas TG and DTG curves are shown in Figure 1 and Figure 2.

**Figure 3:**
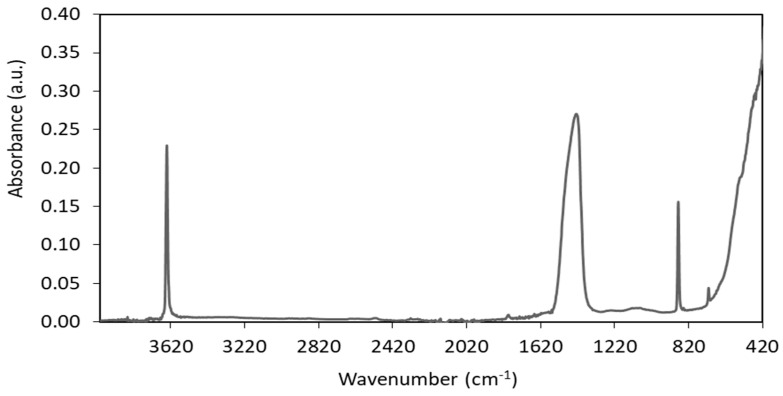
Fourier transformed infrared spectroscopy (FTIR) spectrum of unmodified nanosized CaO.

**Figure 4:**
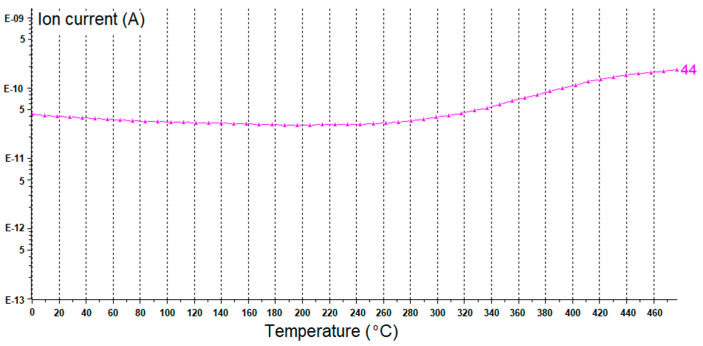
Mass spectrometry (MS) spectrum of unmodified nanosized CaO.

The first mass loss (1.3%) on the TG curve of unmodified nanosized CaO occurred at a temperature below 120 °C and resulted from the desorption of moisture. The next mass loss (19.1%) in the temperature range of 320–500 °C is likely due to the thermal decomposition of the calcium carbonate CaCO_3_, which could be used as a synthesis precursor. On the other hand, exposure of highly reactive surface of nanosized CaO to air during its storage or during preparation of rubber compounds could result in the formation of considerable amount of CO_2_ and H_2_O, which are adsorbed on the surface of CaO in the form of free –OH and carbonate species [45]. The presence of OH groups on the CaO surface was identified by the FTIR, while the presence of carbonate species was confirmed by the FTIR and TG/MS analysis. The results are given in Figure 3 and Figure 4.

Considering the FTIR spectrum of unmodified CaO (Figure 3), the appearance of sharp band at 3641 cm^−1^ may be associated with the stretching and bending vibrations of the hydrogen-bonded -OH groups present on the CaO surface due to physisorbed water [45]. The strong IR absorption band at 1432 cm^−1^ may be attributed to C-O stretching and bending vibrations of CaCO_3_. This band was reported to be characteristic for symmetric stretching vibration of unidentate carbonate [46]. Strong absorption band at 876 cm^−1^ further confirmed the presence of carbonate species [45,46]. The strong IR absorption band at 426 cm^−1^ resulted from the lattice vibrations of CaO [46,47]. The presence of carbonate impurities in the nanosized CaO was confirmed by TG-MS analysis (Figure 4). Broad band in mass spectrum of unmodified CaO was achieved for the mass/charge ratio m/z 44, which could be assigned to CO_2_ resulting from thermal decomposition of carbonate additives. This band appears at a temperature above 320 °C and corresponds to the mass loss on the TG curve of 19.1% occurring in the temperature range of 320–500 °C. Therefore, it can be concluded that the additive present in nanosized CaO is calcium carbonate. CaCO_3_ is commonly used as a chalk in rubber composites. Chalk belongs to the group of inactive fillers, mainly increasing the weight of the rubber composites, while not considerably affecting their performance, crosslink density and cure characteristics. Moreover, it should be noted that the content of carbonate impurity was approx. 0.9 phr in 5 phr of CaO/ALA that was used as a coagent. Considering this, the influence of the carbonate impurity on the crosslinking process and performance of EPM composites may be negligible, especially since the composites are filled with 30 phr of nanosized silica, which is an active filler.

In the TG curves of CaO/ALA decomposition, three stages are present. The first mass loss of 1.0% was observed at a temperature below 120 °C similarly to unmodified CaO. It was likely due to the moisture desorption or removal of the solvent, which remained in the ALA-modified CaO powder. The second mass loss (6.1%) occurred in the temperature range of 200–320 °C and was not observed in the TG curve of raw CaO. Thus, we concluded that this mass loss refers to the decomposition of ALA. The next mass loss of 17.5% at a temperature of 320–500 °C corresponds to the thermal decomposition of the nanosized CaO synthesis precursors, similar to the pure CaO. Determined mass losses were used to calculate the efficiency of CaO modification with ALA. The calculated efficiency of modification process was approximately 75% (Table 5).

### 3.2. Dispersion of CaO/ALA and Silica Particles in the Elastomer

**Figure 5:**
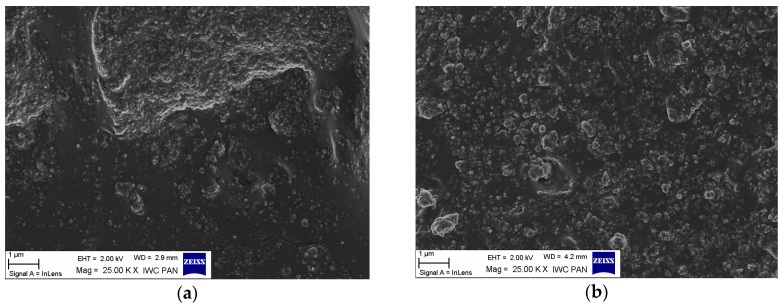

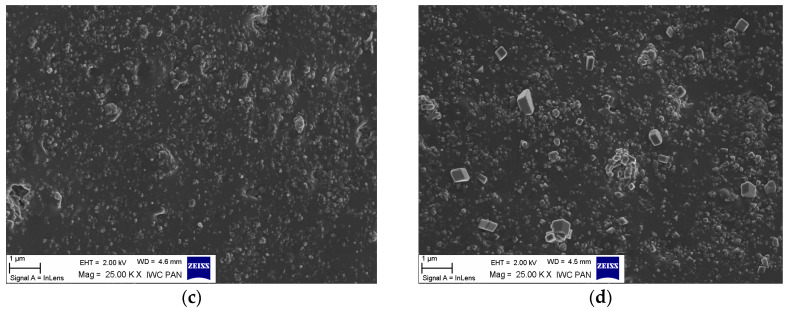
Scanning electron microscopy (SEM) images for silica-filled ethylene–propylene copolymer (EPM) vulcanizates with (**a**) CaO/ALA, (**b**) CaO/ALA and BmimCl, (**c**) CaO/ALA and BmimBF_4_, and (**d**) CaO/ALA and BmimPF_6_.

SEM images were taken to directly examine the distribution of the CaO/ALA coagent and silica nanoparticles in the crosslinked EPM matrix with the presence of ILs (Figure 5a–d). The dispersion of coagent particles in the elastomer matrix is crucial for its activity in crosslinking. Agglomeration decreases the surface area of the coagent and, consequently, the interface between the coagent particles and elastomer chains, whereas agglomeration of the filler particles reduces the reinforcing effect.

For the benchmark containing CaO/ALA (Figure 5a), the nanoparticles of silica and coagent are not homogeneously distributed in the elastomer matrix. The primary nanoparticles create agglomerates with sizes of several µm, which are well wetted by the rubber. The rubber clearly penetrates between the particles forming the agglomerate. ILs promoted the dispersion of CaO/ALA and nanosized silica in the EPM elastomer, and nanoparticles were uniformly distributed in the elastomer matrix (Figure 5b–d). Some agglomerates were observed in the SEM image of the vulcanizate with BmimCl or BmimPF_6_. However, their sizes did not exceed 1 µm, and they exhibited good wettability by the elastomer. The improved dispersion of the coagent and filler particles contributed to the increased crosslink density and enhanced tensile properties of the vulcanizates. The improvement of particle dispersion in the presence of ILs could result from the interactions between ILs and CaO/ALA or silica. Chang et al. [48] demonstrated the hydrogen bonding between the Si-OH and Si-O groups of nanosized silica and the imidazolium cation of ILs. Calcium oxide is a hygroscopic powder. Similar to other oxides, CaO can react with water adsorbed on its surface to form hydroxyl groups, which are considered to be reactive centres [49]. Consequently, the Ca-OH and Ca-O groups located on the surface of the CaO crystals may form hydrogen bonds with the imidazolium cation of ILs similarly to silica. Due to the hydrogen bonding, ILs can be adsorbed on the surface of CaO reducing intermolecular interactions and thus, preventing particles of the coagent from agglomeration. Hydrogen bonding between functional groups on the surface of filler and ILs was also proved to enhance the dispersion of the halloysite nanotubes in the elastomer matrix [43]. No less important is the fact, that ILs can have an antistatic effect, since they are considered to be dispersed and dissociated into ions in the elastomer matrix [50,51]. Another explanation of the positive influence of ILs on the dispersion of coagent in the elastomer matrix could be their lubricating effect, resulting in the surface modification of the nanophase between the particles of coagent and elastomer chains by the ionic liquid [52]. The same effect was reported to promote the dispersion of zinc oxide particles within the polycarbonate matrix [53].

### 3.3. Curing Characteristics and the Crosslink Density of EPM Vulcanizates

**Figure 6:**
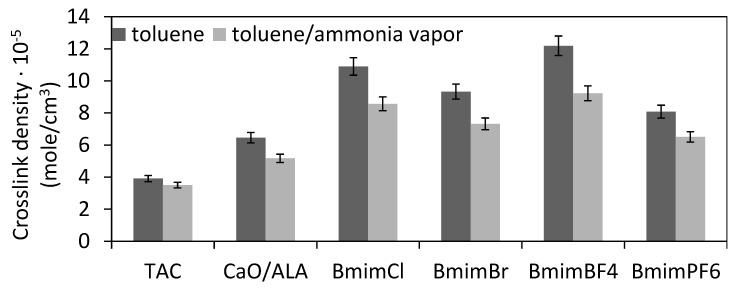
Crosslink densities of silica-filled EPM vulcanizates determined using equilibrium swelling in toluene and toluene with ammonia vapour.

**Figure 7:**
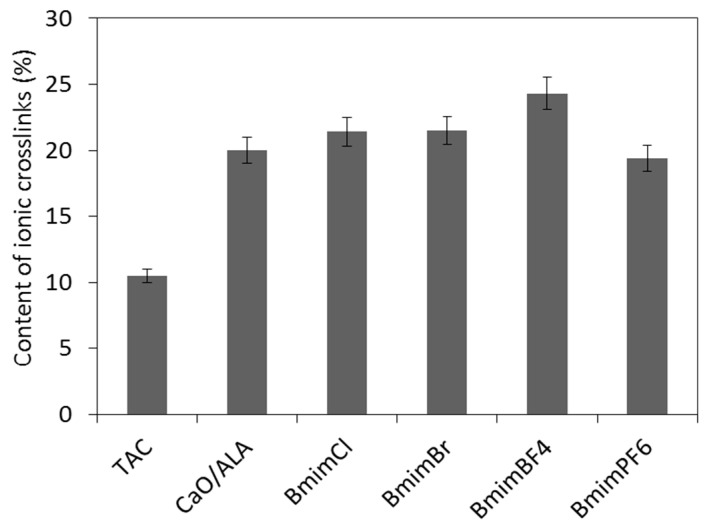
Content of non-covalent crosslinks in silica-filled EPM vulcanizates.

The influence of CaO/ALA and ILs on the cure characteristics of silica-filled EPM compounds and the crosslink densities of the vulcanizates were estimated based on rheometer tests and equilibrium swelling measurements. The results are presented in Table 6 and Figure 6 and Figure 7.

Applying CaO/ALA as a coagent resulted in a higher value of minimum rheometric torque G_min_ during vulcanization than the conventional rubber compound with the TAC coagent. Since G_min_ refers to the viscosity of the uncrosslinked rubber compound, it can be supposed that CaO/ALA slightly increased the viscosity of the silica-filled EPM composites. Most ILs had no considerable effect on G_min_; only BmimPF_6_ decreased the minimum torque to a value similar to that of the TAC-containing rubber compound. Thus, the plasticizing effect of the BmimPF_6_ can be postulated. 

The rubber compound with conventional TAC coagent exhibited an increase in torque during vulcanization of about 41 dNm. CaO/ALA seemed to be very effective in crosslinking the EPM copolymer. Its application increased the torque increment by a factor of three compared to the conventional rubber compound. Torque increment is an indirect measure of the elastomer crosslinking degree. Therefore, it can be supposed that CaO/ALA will effectively increase the degree of elastomer crosslinking. Applying ILs increased the torque increment by more than 20 dNm, so ILs seem to increase the efficiency of crosslinking reactions. Only BmimPF_6_ did not considerably affect ΔG during vulcanization. It should be noted, that BmimPF_6_ demonstrates the highest thermal stability among the used ILs [54]. Owing to the highest electronegativity of the anion, BmimPF_6_ is also the most chemically stable with respect to reaction [55]. Hence, BmimPF_6_ is expected to have the least influence on the course and efficiency of the crosslinking reactions among the studied ILs. 

CaO/ALA and ILs had no influence on the scorch time, which determines the safety of EPM processing at a given temperature. No reduction in the scorch time was achieved, which is important for technological reasons. The optimal vulcanization time of the TAC-containing rubber compound was 8 min. Applying CaO/ALA extended the optimal vulcanization time of the EPM by 6 min but achieved a much higher crosslinking degree. The vulcanization time for the composites containing ILs was in the range of 14 to 16 min. The influence of the anion present in the ionic liquid was not observed. 

Based on the cure characteristics of the EPM compounds, we concluded that CaO/ALA and ILs increased the crosslinking degree of the elastomer, likely due to the formation of additional ionic crosslinks with the use of functional groups of the coagents. Thus, to confirm this conclusion, the crosslink density of the vulcanizates was estimated based on their equilibrium swelling in toluene, as well as in toluene in a desiccator with saturated ammonia vapor. The results are given in Figure 6 and Figure 7.

Most importantly, application of CaO/ALA considerably increased the crosslink density of the EPM vulcanizates compared to those produced using TAC (Figure 6). ILs, especially BmimBF_4_, caused a further increase in the crosslink density of the vulcanizates. The positive effect of ILs on the crosslink density of EPM may have resulted from the better dispersion of the CaO/ALA particles in the elastomer, which increased contact between the functional groups of the coagent and the elastomer chains, thereby improving the activity of the coagent in the crosslinking reactions. It should be noted, that the highest crosslink density was demonstrated by the vulcanizate containing BmimBF_4_, for which the most uniform distribution of the CaO/ALA particles was achieved. On the other hand, the lowest crosslink density was exhibited by EPM vulcanizates with BmimPF_6._ This may result from the worse dispersion of the coagent particles in the elastomer matrix containing this ionic liquid and the aforementioned high-thermal and -chemical stability of BmimPF_6_. Additionally, ILs are believed to catalyze the interfacial crosslinking reactions, as reported for the peroxide crosslinking of a hydrogenated acrylonitrile–butadiene elastomer [16] and for the sulfur vulcanization of unsaturated rubbers [35,39,40].

It should be noted that the crosslink density of the EPM vulcanizates was improved by the formation of additional non-covalent coagent links in the presence of CaO modified with unsaturated acid (Figure 7).

Considering the results presented in Figure 7, a non-covalent link content (approximately 11%) determined for the vulcanizate with a TAC coagent was likely due to interactions at the rubber–silica interface [6,7,13]. The CaO/ALA did not affect the amount of linkages between silica particles and elastomer chains. Therefore, the content of non-covalent crosslinks in the EPM elastomer network formed by the hybrid coagent was estimated as the number of non-covalent links in the vulcanizates containing a CaO/ALA coagent reduced by the number of the links at the silica–elastomer interface, determined for TAC-containing EPM vulcanizate. For the possible mechanism of the crosslinking reaction in the presence of coagents based on metal oxide modified with unsaturated organic acid, which is presented in [14], these crosslinks are likely labile ionic clusters. The content of non-covalent crosslinks in the CaO/ALA-containing elastomer was approximately 20%. ILs slightly increased the content of ionic crosslinks in the EPM vulcanizates. The highest content of ionic crosslinks was achieved for the vulcanizate containing BmimBF_4_, for which the most homogeneous distribution of the CaO/ALA particles was observed. On the other hand, the lowest crosslink density and the smallest content of ionic crosslinks were determined for the vulcanizate with BmimPF_6_, which was characterized by a less uniform dispersion of the coagent and silica particles than the composites with other ILs. Thus, homogeneous dispersion of coagent increases the interface between its particles and consequently functional groups, and elastomer chains improving the efficiency of crosslinking. 

**Figure 8:**
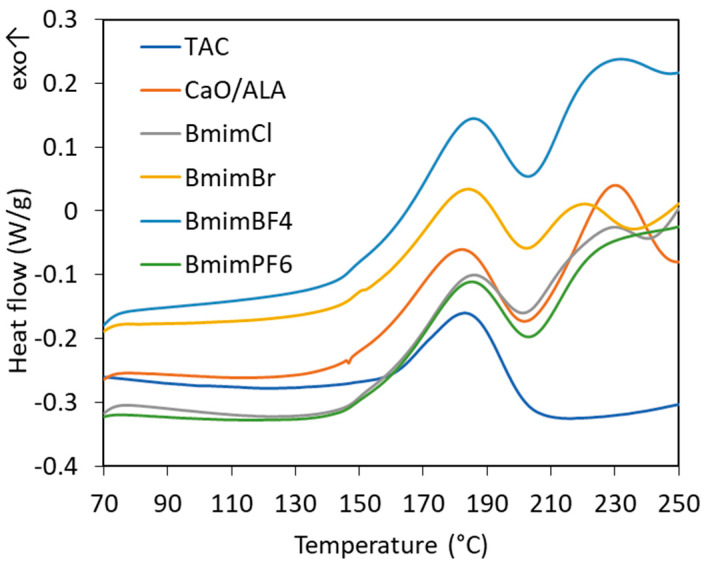
Differential scanning calorimetry (DSC) curves for the vulcanization of silica-filled EPM containing CaO/ALA and ILs.

After studying the effects of CaO/ALA and ILs on the curing characteristics of EPM, their influence on the temperature range and enthalpy of vulcanization was then examined using DSC analysis. The results for EPM compounds are presented in Table 7 and Figure 8.

The vulcanization of the EPM with TAC as coagent is a one-step exothermic process that occurs in a temperature range of 145–200 °C with an enthalpy of approximately 22 J/g (Figure 8). According to Dikland et al., during this process, peroxide crosslinking occurs alongside the covulcanization of the TAC coagent domains with the surrounding elastomer matrix. Irreversible coagent bridges are formed as a result [56]. On the other hand, the vulcanization of EPM with CaO/ALA as a coagent is a two-step process. The first step occurs in a temperature range of 147–199 °C, with an enthalpy of approximately 23 J/g, which is similar to the rubber compound containing TAC as a coagent. The second step of vulcanization takes place in a temperature range of 208–246 °C and was observed for all rubber compounds containing CaO/ALA. Thus, we concluded that during this step, crosslinking occurred due to the formation of noncovalent ionic links between the functional groups of coagent and EPM elastomer chains. The ILs had no significant influence on the temperature of both steps of vulcanization compared to that of the EPM containing a conventional TAC coagent. Analyzing the differences between rubber compounds containing particular ILs, the highest onset vulcanization temperature was determined for EPM containing BmimPF_6_, so the most chemically and thermally stable ionic liquid, which thus is supposed to have the least influence on the efficiency of the crosslinking reactions. It is worth noting that the ILs did not considerably affect the enthalpy of the first step of vulcanization or the peroxide crosslinking but significantly reduced the enthalpy of the second step featuring the formation of ionic crosslinks. This is important for technological reasons because fewer exothermic processes are safer and easier to control than more exothermic processes.

### 3.4. Mechanical Properties of EPM Vulcanizates

The aim of applying the ILs was also to achieve a homogeneous dispersion of the CaO/ALA coagent and silica nanoparticles in the elastomer and thereby improve the mechanical properties of the vulcanizates. The mechanical properties of the EPM vulcanizates are given in Table 8.

Vulcanizates with CaO/ALA and ILs exhibited an approximately 2.6–4.3 MPa higher SE_300_ modulus compared to the vulcanizate cured with TAC. This resulted from their significantly higher crosslink densities. The highest SE_300_ module was observed for the vulcanizate with the highest crosslink density, i.e., the one containing BmimBF_4_. The EPM vulcanizate with TAC as a coagent demonstrated a tensile strength of 11.4 MPa and an elongation at break of 783%. CaO/ALA improved the TS of the vulcanizate by 4.4 MPa. The EB values were reduced by more than 350% due to the higher crosslink density of the vulcanizate. The ILs enhanced the TS of the vulcanizates without a significant influence on their elongation at break. This impact was the most pronounced for the BmimBF_4_–containing elastomer (TS of approximately 21 MPa), which demonstrated the most uniform distribution of the filler and coagent particles in the crosslinked elastomer matrix resulting in the highest crosslink density of the vulcanizate and the highest content of non-covalent links that are labile ionic crosslinks. Ionic clusters are known to possess the ability to move or rearrange within the elastomer network under the influence of external stress increasing the tensile strength of vulcanizates [57].

### 3.5. Weather Aging Resistance of EPM Vulcanizates

**Figure 9:**
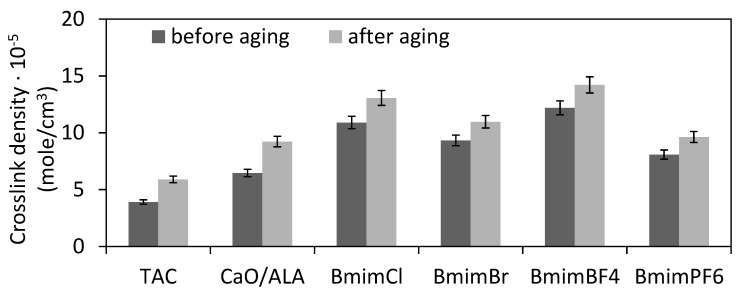
Crosslink densities of silica-filled EPM vulcanizates after weather aging.

**Figure 10:**
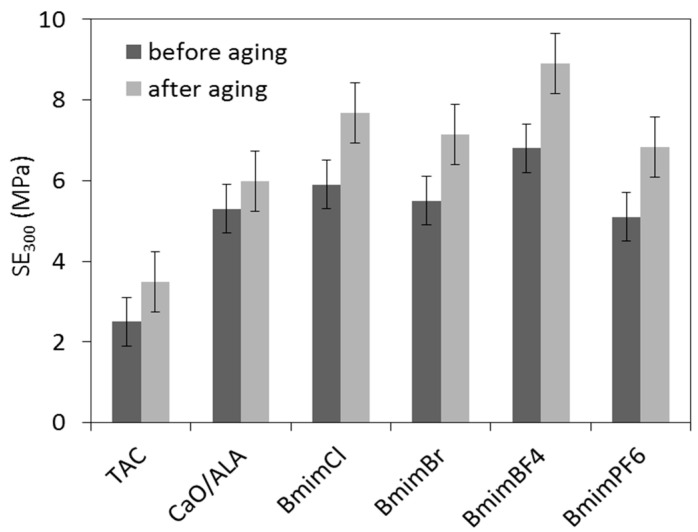
Modulus at 300% relative elongation of silica-filled EPM vulcanizates after weather aging.

**Figure 11:**
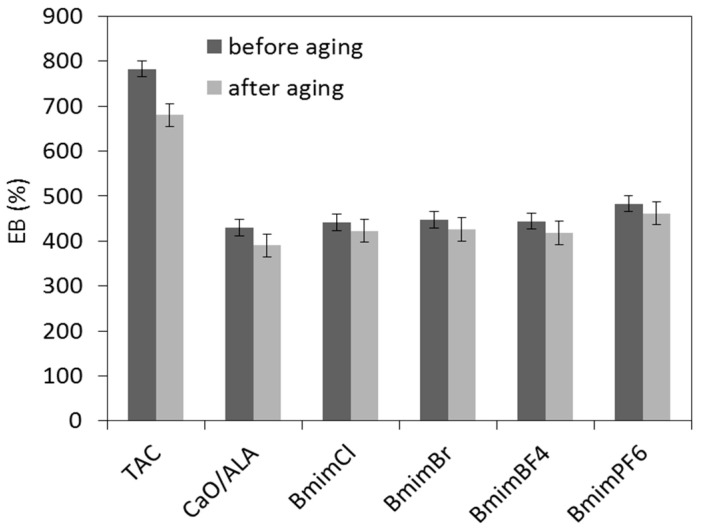
Elongation at break of silica-filled EPM vulcanizates after weather aging.

**Figure 12:**
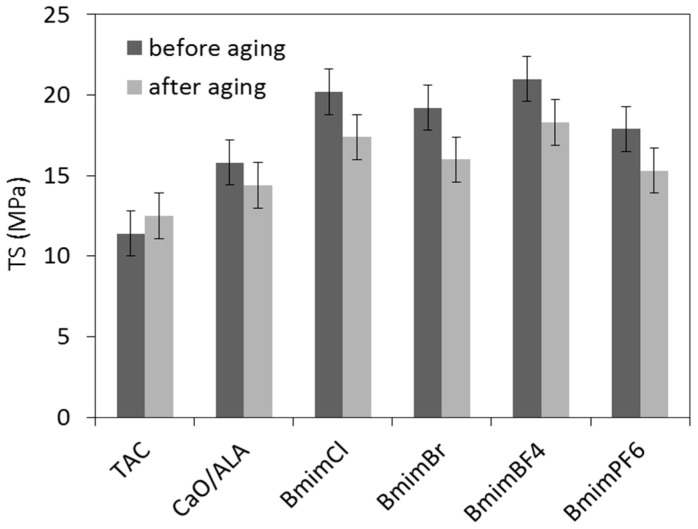
Tensile strength of silica-filled EPM vulcanizates after weather aging.

Aging resistance is crucial property of elastomer products dedicated for outdoor applications. Sunlight, moisture and high temperatures cause aging of rubber products and consequently deteriorate their mechanical performance. Therefore, the influence of the CaO/ALA coagent and ILs on vulcanizate resistance to weather aging was investigated. The effects of weather aging on the mechanical performance and crosslink density of EPM vulcanizates are presented in Figure 9, Figure 10, Figure 11 and Figure 12.

Weather aging factors initiated further crosslinking of the elastomer. Similar increases in the crosslink density due to aging were observed for all vulcanizates, including the benchmark with the TAC coagent. Regarding the vulcanizates with ILs, the changes in the crosslink densities were similar considering the standard deviation of the measured parameters (Figure 9). 

Weather aging caused the SE_300_ modulus to increase, especially for vulcanizates containing CaO/ALA with ILs (Figure 10), whereas the EB values were reduced by approximately 100% for vulcanizate with TAC and by 20–50% for vulcanizates with CaO/ALA and ILs compared to the samples before aging (Figure 11). It was due to the increase in the crosslink density of the vulcanizates. ILs reduced the influence of weather aging on the EB of the vulcanizates. 

Due to the increase in the crosslink density, the aging process deteriorated the tensile strength of the vulcanizates containing CaO/ALA separately and together with ILs (Figure 12). The values of the TS were approximately 2–3 MPa lower than those for the vulcanizates before the aging procedure. However, a slight improvement in the TS was achieved for the vulcanizate containing TAC. It should be noted that this vulcanizate showed a significantly lower crosslink density before aging compared to the vulcanizates with CaO/ALA and ILs. It is commonly known that TS increases with the number of crosslinks to a certain value of the crosslink density. After exceeding the optimal value of this parameter, a further increase in the crosslink density leads to a decrease in TS. Therefore, it can be concluded that before the aging process, the vulcanizate with TAC presented a crosslink density lower than the optimal value. Thus, further crosslinking resulted in improvement of the TS. On the other hand, the crosslink densities of the vulcanizates with CaO/ALA seemed to be higher than optimal. Therefore, the further crosslinking that occurred during aging caused deterioration of the TS. 

To quantitatively estimate the changes in tensile properties (TS and EB) due to aging, the aging coefficient A_F_ was calculated (Table 9). The values of the A_F_-factor that are closer to 1 indicate smaller changes in the mechanical properties resulting from the aging process and, consequently, better resistance of the material to aging.

Due to its stable saturated backbone, the EPM elastomer is resistant to weather, as confirmed by the A_F_-coefficient 0.95 for the benchmark containing TAC. The application of the CaO/ALA coagent slightly reduced the aging factor to a value 0.83. The ILs did not affect the resistance of the EPM to weather aging compared to the CaO/ALA-containing vulcanizate. The values of the A_F_-coefficient for vulcanizates containing ILs were in the range of 0.80–0.82. It can be concluded that the vulcanizates containing CaO/ALA and ILs had good resistance to weather aging process.

### 3.6. Thermal Stability of EPM Vulcanizates

**Figure 13:**
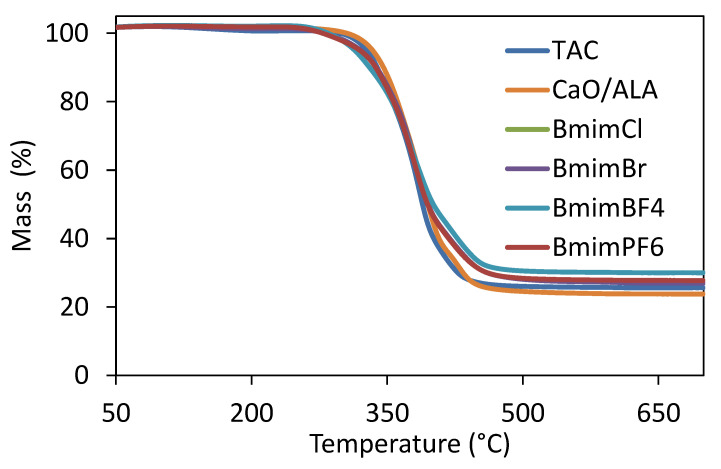
TG curves of EPM vulcanizates.

**Figure 14:**
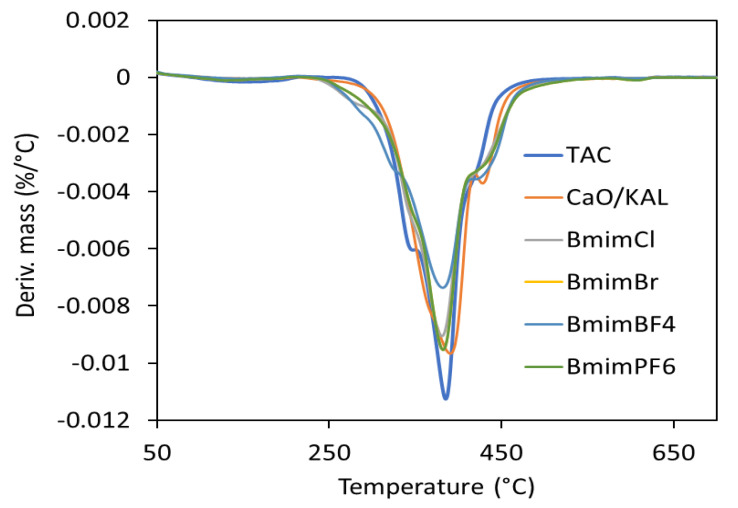
DTG curves of EPM vulcanizates.

A TG analysis was performed to investigate the effects of CaO/ALA and ILs on the thermal stability of the EPM vulcanizates. The results are given in Table 10 and Figure 13 and Figure 14.

The TG and DTG curve analyses showed that the samples with TAC and CaO/ALA had similar thermal stability (Figure 13 and Figure 14). The TG curves almost overlap. No mass loss occurred until reaching a temperature of approximately 320 °C. Above this temperature, pyrolysis of the EPM elastomer began, together with the thermal decomposition of organic ingredients, such as dicumyl peroxide, TAC, and ALA grafted on the surface of the CaO. The peak of the DTG curve at a temperature of about 390 °C results from the pyrolysis of the EPM elastomer, crosslinking agent, and coagent. Applying ILs deteriorated the thermal stability of the EPM vulcanizates. The onset decomposition temperature (T_5%_) was reduced by 14–18 °C compared to the vulcanizate with CaO/ALA. This was due to the thermal decomposition of the ILs, which preceded the pyrolysis of the EPM elastomer. The thermal stability of ILs depends mainly on the nature of anion. For ILs with Bmim cation, the thermal stability increases in the following order: chloride, bromide tetrafluoroborate and hexafluorophosphate [54], and the differences between the onset decomposition temperature of these ILs are significant, especially between chloride or bromide and tetrafluoroborate or hexafluorophosphate. Considering the differences between the T_5%_ of the EPM vulcanizates with studied ILs, no significant effect of the ionic liquid anion on the thermal stability was observed, taking into account the measurement error. Moreover, the thermal stability of the vulcanizates did not follow the order of the thermal stability of the ILs. It may result from the small amount of the ILs (3 phr) in the vulcanizate compared to other ingredients (e.g., 100 phr of EPM, 30 phr of silica), which determined the thermal stability of the composite and masked the influence of the ILs structure. Since the measurements were performed in an inert atmosphere, the residue after thermal decomposition at 700 °C was approximately 3% higher for the vulcanizates with ILs. Pyrolysis of the ILs left an additional solid residue enriched in carbon, which was derived from the carbon skeleton of the ILs. From a practical point of view, such a reduction in thermal stability under the influence of ILs should not reduce the potential applications of EPM products.

## 4. Conclusions

A new coagent for EPM peroxide crosslinking was studied. This hybrid coagent based on nanosized CaO modified with unsaturated carboxylic acid (i.e., allylmalonic acid) was developed to provide non-covalent crosslinks in the elastomer network, in the form of labile ionic aggregates that are capable of sliding on the surfaces of solid CaO particles. ILs with 1-butyl-3-methylimidazolium cation were applied to improve the dispersion degree of the filler and CaO/ALA nanoparticles in the crosslinked elastomer matrix.

Application of the CaO/ALA slightly increased the optimal vulcanization time compared to the conventional TAC coagent. Most importantly, the reaction of the CaO/ALA coagent functional groups with the rubber chains considerably increased the crosslink density of the vulcanizates due to the formation of ionic crosslinks. ILs did not affect the optimal vulcanization time but significantly improved the crosslink density of the vulcanizates. This may result from the better dispersion of the CaO/ALA particles in the elastomer, which increased the contact between the functional groups of the coagent and the EPM rubber chains, consequently improving the activity of the coagent in the crosslinking reactions. The highest crosslink density demonstrated the vulcanizates containing BmimBF_4_, for which the most uniform dispersion of the coagent and filler particles in the EPM matrix was observed. CaO/ALA increased the tensile strength and considerably reduced the elongation at break in comparison with the vulcanizate containing TAC. ILs resulted in further improvement in the TS of the EPM vulcanizates without a significant influence on their elongation at break. The highest TS showed the vulcanizates with BmimBF_4_. We concluded, that two main factors contributed to improving the tensile strength of the vulcanizates. The first is the increase in crosslink density due to the action of the CaO/ALA coagent, while the second is the formation of ionic crosslinks in the elastomer network and their labile nature.

CaO/ALA slightly reduced the resistance of the EPM to weather aging, whereas ILs did not affect this property compared to the CaO/ALA-containing vulcanizate. On the other hand, CaO/ALA had no influence on the thermal stability of the EPM vulcanizates. Applying ILs reduced the onset temperature of thermal degradation by approximately 18 °C compared to the conventional vulcanizate with TAC. However, taking into account the range of changes in the vulcanizates aging resistance, and thermal stability, it can be concluded that CaO/ALA and ILs will not reduce the potential applications of EPM composites. Most importantly, CaO/ALA and ILs allow one to improve crosslink density and, consequently, the mechanical properties of the EPM vulcanizates obtained by peroxide crosslinking.

## Figures and Tables

**Table 1:** Molecular mass and the structure of allylmalonic acid (ALA).

Molar mass (g/mole)	Molecular Structure
144.12	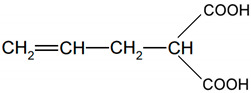

**Table 2:** Ionic liquids (ILs) used in this study.

ILs	Molar Mass (g/mole)	Molar Content (mmole/100 g of EPM)
1-butyl-3-methylimidazolium chloride (BmimCl)	174.7	8.6
1-butyl-3-methylimidazolium bromide (BmimBr)	219.1	7.2
1-butyl-3-methylimidazolium tetrafluoroborate (BmimBF_4_)	226.0	6.7
1-butyl-3-methylimidazolium hexafluorophosphate (BmimPF_6_)	284.2	5.3

**Table 3:** General recipe of the ethylene–propylene copolymer (EPM) compounds, parts per hundred of rubber (phr) (R1 and R2 (benchmark samples); EPM1-4 (rubber compounds containing the ILs presented in Table 2)).

Ingredient (phr)	R1	R2	EPM1-4
Ethylene–propylene copolymer (EPM)	100	100	100
Dicumyl peroxide (DCP)	4	4	4
Silica Aerosil 380	30	30	30
Triallyl cyanurate (TAC)	1	-	-
CaO/ALA	-	5	5
Ionic liquid	-	-	1.5

**Table 4:** Mass losses obtained from thermogravimetric (TG) curves for pure and ALA-modified CaO (standard deviation: ∆m ± 0.9%).

Sample	Δm 25–200 °C (%)	Δm 200–320 °C (%)	Δm 320–500 °C (%)	Residue at 500 °C (%)
CaO	1.3	-	19.1	79.6
CaO/ALA	1.0	6.1	17.5	75.4

**Table 5:** Content of ALA in the coagent and the efficiency of CaO modification.

Sample	Amount of ALA Used for CaO Modification (mmole/g of CaO)	Amount of ALA in Modified CaO (mmole/g of CaO)	Efficiency of Modification (%)
CaO/ALA	0.56	0.42	75.0

**Table 6:** Cure characteristics of silica-filled EPM compounds at 160 °C (G_min_—minimum torque; ΔG—increment of torque during vulcanization; t_90%_—optimal vulcanization time; t_20%_—scorch time; standard deviations: G_min_ ± 2.5, ∆G ± 3.1 dNm, t_20%_ ± 0.4 min, and t_90%_ ± 0.9 min).

Rubber Compound		G_min_ (dNm)	ΔG (dNm)	t_20%_ (min)	t_90%_ (min)
R1	TAC	35.5	41.2	1.2	8
R2	CaO/ALA	43.4	122.9	1.3	14
EPM1	BmimCl	44.0	148.1	1.5	15
EPM2	BmimBr	43.2	141.7	1.5	16
EPM3	BmimBF_4_	43.5	153.8	1.1	14
EPM4	BmimPF_6_	35.9	124.9	1.6	14

**Table 7:** Temperature range and enthalpy (ΔH) of vulcanization measured by differential scanning calorimetry (DSC) for silica-filled EPM compounds (standard deviations: temperature ±3 °C; ∆H ± 2.2 J/g).

Rubber Compound		Vulcanization Temperature (°C)		Vulcanization Enthalpy (J/g)	
		1st Step	2nd Step	1st Step	2nd Step
R1	TAC	145–200	-	21.8	-
R2	CaO/ALA	147–199	208–246	23.0	21.8
EPM1	BmimCl	142–199	205–239	17.5	7.4
EPM2	BmimBr	141–200	205–239	20.1	5.6
EPM3	BmimBF_4_	140–201	207–247	22.5	10.6
EPM4	BmimPF_6_	145–200	206–242	18.8	8.7

**Table 8:** Mechanical properties of silica-filled EPM vulcanizates (SE_300_—modulus at a relative elongation of 300%, TS—tensile strength, EB—elongation at break, standard deviations: SE_300_ ± 0.6 MPa, TS ± 1.4 MPa, EB ± 18%).

Vulcanizate		SE_300_ (MPa)	TS (MPa)	EB (%)
R1	TAC	2.5	11.4	783
R2	CaO/ALA	5.3	15.8	430
EPM1	BmimCl	5.9	20.2	442
EPM2	BmimBr	5.5	19.2	447
EPM3	BmimBF_4_	6.8	21.0	444
EPM4	BmimPF_6_	5.1	17.9	483

**Table 9:** Weather aging coefficient (A_F_) of silica-filled EPM vulcanizates (standard deviation A_F_ ± 0.04).

Vulcanizate		A_F_ (-)
R1	TAC	0.95
R2	CaO/ALA	0.83
EPM1	BmimCl	0.82
EPM2	BmimBr	0.80
EPM3	BmimBF_4_	0.82
EPM4	BmimPF_6_	0.82

**Table 10:** Decomposition temperature at 5% of the mass change (T_5%_), DTG peak temperature (T_DTG_), and total mass loss (Δm) during decomposition of the EPM vulcanizates (standard deviations: T_5%_, T_DTG_ ± 3 °C; Δm ± 1.5%).

Vulcanizate		T_5%_ (°C)	T_DTG_ (°C)	Δm (%)	Residue at 700 °C (%)
R1	TAC	324	390	76.3	23.7
R2	CaO/ALA	328	394	77.2	22.8
EPM1	BmimCl	314	385	74.2	25.8
EPM2	BmimBr	314	386	74.9	25.1
EPM3	BmimBF_4_	310	386	73.4	26.4
EPM4	BmimPF_6_	312	385	73.9	26.1
